# Effect of gamma irradiation on filtering facepiece respirators and SARS-CoV-2 detection

**DOI:** 10.1038/s41598-021-99414-6

**Published:** 2021-10-06

**Authors:** Khaled Al-Hadyan, Ghazi Alsbeih, Najla Al-Harbi, Sara Bin Judia, Maha Al-Ghamdi, Akram Almousa, Ibtihaj Alsharif, Razan Bakheet, Khaldoun Al-Romaih, Maha Al-Mozaini, Salem Al-Ghamdi, Belal Moftah, Rashed Alhmaid

**Affiliations:** 1 Biomedical Physics Department, King Faisal Specialist Hospital and Research Centre (KFSH&RC), Riyadh, Saudi Arabia; 2 Infection and Immunity Department, KFSH&RC, Riyadh, Saudi Arabia; 3 Translational Genomics Department, KFSH&RC, Riyadh, Saudi Arabia; 4 Infection Control and Hospital Epidemiology Department, KFSH&RC, Riyadh, Saudi Arabia; 5 General Corporate Consultancy Department, KFSH&RC, Riyadh, Saudi Arabia

**Keywords:** Viral infection, Infection

## Abstract

To cope with the shortage of filtering facepiece respirators (FFRs) during the coronavirus (COVID-19) pandemic, healthcare institutions were forced to reuse FFRs after applying different decontamination methods including gamma-irradiation (GIR). The aim of this study was to evaluate the effect of GIR on the filtration efficiency (FE) of FFRs and on SARS-CoV-2 detection. The FE of 2 FFRs types (KN95 and N95-3 M masks) was assessed at different particle sizes (0.3–5 µm) following GIR (0–15 kGy) delivered at either typical (1.65 kGy/h) or low (0.5088 kGy/h) dose rates. The detection of two SARS-CoV-2 RNA genes (E and RdRp4) following GIR (0–50 kGy) was carried out using RT-qPCR assay. Both masks showed an overall significant (*P* < 0.001) reduction in FE with increased GIR doses. No significant differences were observed between GIR dose rates on FE. The GIR exhibited significant increases (*P* ≤ 0.001) in the cycle threshold values (ΔCt) of both genes, with no detection following high doses. In conclusion, complete degradation of SARS-CoV-2 RNA can be achieved by high GIR (≥ 30 kGy), suggesting its potential use in FFRs decontamination. However, GIR exhibited adverse effects on FE in dose- and particle size-dependent manners, rendering its use to decontaminate FFRs debatable.

## Introduction

The filtering facepiece respirators (FFRs), including N95 masks, play an important role in infection prevention and control by reducing the airborne transmission of infectious illnesses from patients to health practitioners^1,2^. The N95 mask is a high efficacy single-use FFR that can block up to 95% of particles of ≥ 0.3 µm in diameter. The N95 mask is approved by the United States Food and Drug Administration (FDA) and the National Institute for Occupational Safety and Health (NIOSH) for use as personal protective equipment (PPEs) in medical settings^1,2^.

Due to the global shortage of N95 masks during the outbreak of Coronavirus Disease (COVID-19) caused by the emerging severe acute respiratory syndrome coronavirus 2 (SARS-CoV-2), NIOSH has issued a recommended guidance for the extended use and limited reuse of N95 masks in emergency medical settings^3^. Although the NIOSH guidance can significantly reduce the consumption of N95 masks during the pandemic, concerns about these policies have been raised^4,5^. These concerns are related to the long survival of the SARS-CoV-2 virus on the outer layer of N95 masks, leading to cross-contamination between staff and patients^4,5^. Therefore, decontamination and subsequent reuse of the N95 masks were highly recommended to cope with the expected shortage in such a pandemic.

There are six well-characterized N95 decontamination procedures including vapor hydrogen peroxide (VHP), ethylene oxide, moist heat incubation, microwave oven, ultraviolet germicidal irradiation (UVGI), and gamma irradiation (GIR)^6–10^. Although the USA Centers for Disease Control (CDC) did not approve the routine decontamination of N95 masks, it released emergency guidelines on the N95 decontamination methods and indicated that UVGI, VHP and moist heat had shown the most promising results^7^. However, these three methods have certain logistical and technical limitations including the small capacity, the limited penetration and the high risk of pathogen cross-contamination should the used FFRs be removed from their biosafety container/bag and handled individually during the decontamination process^6–10^.

Hypothetically, GIR has several advantages among other decontamination methods such as better penetration, better certainty of sterility and independence from temperature and pressure conditions^11^. However, concerns have been raised regarding the adverse effect of GIR on the physical properties of FFRs materials, in particular polymer fabric, a major component in manufacturing FFRs^12–15^. Briefly, the FFRs filter fabrics are made mainly by polypropylene (PP), a thermoplastic polymer used in a wide variety of applications, in addition to other secondary components such as nylon, cotton and polyester^14^. The GIR was shown to cause substantial adverse alterations in the physical characteristics of PP, such as enhanced polymer oxidation, polymer chain scission or cross-linking, which can degrade the PP mechanical filtration performance and its clinical protection^12,13,15^. Regardless of these concerns, recent studies during the COVID-19 pandemic reconsider the potential deployment of GIR in the decontamination and reuse of the FFRs^6,16–19^.

Since 1971, GIR has been used to inactivate many types of microorganisms, including single-stranded RNA viruses^20,21^. The mechanism of virus inactivation using GIR is thought to fall into direct and indirect categories^22–24^. Direct virus inactivation is mainly caused by radiolytic cleavage or cross-linking of genetic material. Indirect virus inactivation is caused by the oxidative damage of genetic material caused by free radicals issued from the radiolysis of water. The major molecular target of both mechanisms is believed to be the nucleic acids, whereas radiation damages to proteins and lipids in the viral envelope are believed to play a minor role in the virus inactivation^25–27^.

The viruses of the corona family such as SARS-CoV (including SARS-CoV-2) and Middle East respiratory syndrome coronavirus (MERS-CoV) were reported to be inactivated by a dose of 10 kGy, although lower doses have not been extensively studied^28–31^. Only one study reported that a dose of 0.15 kGy, a dose not expected to inactivate viruses, was insufficient to inactivate active cultured SARS-CoV^32^.

To our knowledge, no attempt has been made so far to study the effect of low dose rate GIR on either SARS-CoV-2 activity or the filtration efficiency (FE) of FFRs. Therefore, the aim of this study was to evaluate the effect of typical and low dose rates GIR on the FE of two common FFR types, KN95 and 3M-N95 masks, hypothesizing that the eventual adverse effect of GIR on FFRs could be minimized by using low dose rate GIR. Simultaneously, the study explored the capability of low dose rate GIR to sterilize infected FFRs by evaluating the detectability of SARS-CoV-2 RNA following gamma-irradiation using reverse transcription quantitative real-time polymerase chain reaction (RT-qPCR) assay.

## Results

### Initial filtration efficiency (FE) of FFRs

**Figure 1:**
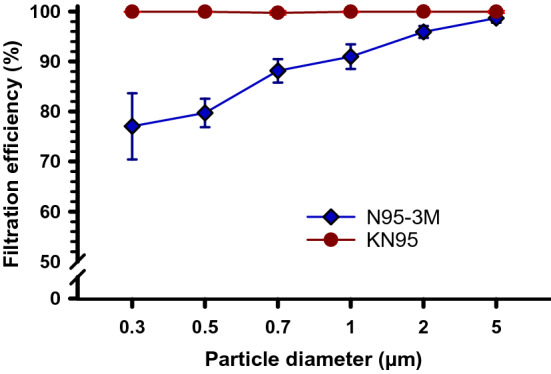
Filtration efficiency (FE) of KN95 and N95-3M FFRs. KN95 (n = 10) and N95-3M (n = 10) FFRs were applied to FE evaluation for different particle sizes (0.3–5 µm) using AeroTrak particle counter. Symbols represent the mean and error bars indicate the standard deviation. The overall FE of KN95 masks was significantly higher (*P* = 0.022) than N95-3M masks. At each particle size, the KN95 masks’ FE was significantly higher (*P* ≤ 0.001) than N95-3M masks.

0The results of the initial FE (before any treatment) of KN95 and N95-3M masks are presented in Fig. 1. The KN95 masks exhibited constantly higher FE (≥ 99.74%) at all particle sizes (PSs) than the N95-3M masks (77.1–98.7%). Although the FE of the N95-3M mask increased progressively with increasing the PSs, it showed mediocre (≤ 91.0%) FE at small PSs (0.3–1 µm) compared to larger particles (2 and 5 µm) which showed satisfactorily FE between 95.9 and 98.7%. Statistically, the paired t-test showed that the overall mean FE (at all particle sizes) of KN95 was significantly higher than N95-3M masks (two-tailed *P*-value = 0.022). Furthermore, the t-test showed that the individual FE at each PS was significantly higher (*P* ≤ 0.001) for KN95 compared to N95-3M masks.

### Filtration efficiency (FE) of FFRs following gamma irradiation (GIR)

**Figure 2:**
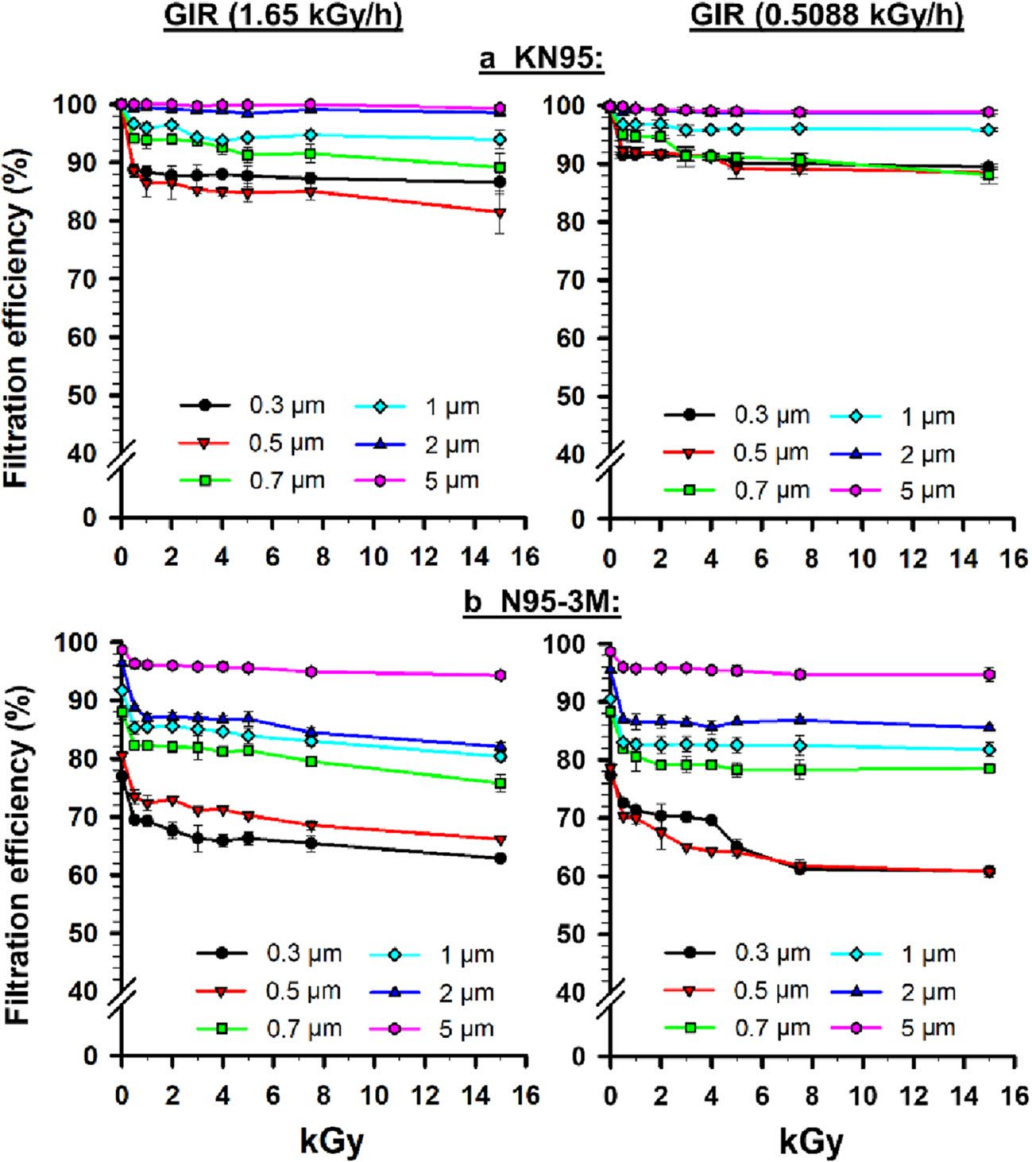
Filtration efficiency (FE) of a. KN95 and b. N95-3M masks for different particle sizes (0.3–5 µm) following gamma irradiation (GIR) at either typical (1.65 kGy/h) or low (0.5088 kGy/h) dose rates. Symbols represent the mean and error bars indicate the standard error. Statistically, both masks showed an overall significant (*P* < 0.001) reduction in FE with increased radiation doses, for both dose rates. Pairwise comparisons for KN95 masks irradiated with typical gamma radiation: particle sizes of 0.3, 0.5, 0.7 and 1 µm versus 2 and 5 µm were significant (*P* ≤ 0.016); particle sizes of 0.3 and 0.5 µm versus 0.7 and 1 µm were significant (*P* ≤ 0.002). Pairwise comparison for KN95 masks irradiated with low gamma radiation: 0.3 µm versus 5 µm *P* = 0.001; 0.5 µm versus 5 µm *P* = 0.002; 0.7 µm versus 5 µm *P* = 0.002; 0.3 µm versus 2 µm *P* = 0.014; 0.5 µm versus 2 µm *P* = 0.020; 0.7 µm versus 2 µm *P* = 0.020. Pairwise comparison for N95-3M masks irradiated with typical gamma radiation: particle sizes of 0.3, 0.5, 0.7, 1 and 2 µm vs. 5 µm were significant (*P* < 0.001); particle sizes of 0.3, 0.5, 0.7 and 1 µm versus 2 µm were significant (*P* ≤ 0.002); particle sizes of 0.3, 0.5 and 0.7 µm versus 1 µm were significant (*P* < 0.001); 0.3 and 0.5 µm versus 0.7 µm were significant (*P* < 0.001); 0.3 µm versus 0.5 µm *P* < 0.001. Pairwise comparison for N95-3 M masks irradiated with low gamma radiation: particle sizes of 0.3, 0.5, 0.7, 1 and 2 µm versus 5 µm were significant (*P* < 0.001); particle sizes of 0.3, 0.5, 0.7 and 1 µm versus 2 µm were significant (*P* ≤ 0.005); particle sizes of 0.3 and 0.5 µm versus 0.7 and 1 µm were significant (*P* < 0.001).

The FE of KN95 and 3M-N95 masks was assessed following typical (1.65 kGy/h) and low (0.5088 kGy/h) dose rates GIR at PSs of 0.3, 0.5, 0.7, 1, 2 and 5 µm. Results are presented in Fig. 2. The FE displayed dose- and size-dependent decline for 0.3, 0.5, 0.7, 1 and 2 µm particles sizes, with some particularities for each mask type and dose rate. As for the 5 µm PS, the FE showed little or no decrease with increasing GIR dose.

#### Filtration efficiency (FE) of FFRs following typical dose rate gamma irradiation

The highest decreases in FE of KN95 and N95-3M masks were observed following 15 kGy at PSs of 0.5 and 0.3 µm, with percentage decreases of 18.5% and 18.2%, respectively (Supplementary Table S1). Statistically, the average FE values of KN95 masks were significantly lower than control at GIR doses of 4, 5 and 15 kGy (One Way Repeated Measure Analysis of Variance, *P* ≤ 0.030), whereas the average FE values of N95-3M masks were significantly lower than control at GIR dose of 4, 5, 7.5 and 15 kGy (*P* ≤ 0.025, Table 1).

**Table 1:** Filtration efficiency of KN95 and N95-3M masks before and after gamma radiation dose of 0–15 kGy.

Dose (kGy)	KN95				N95-3M			
	Typical dose rate		Low dose rate		Typical dose rate		Low dose rate	
	FE average*	*P* value	FE average*	*P* value	FE average*	*P* value	FE average*	*P* value
0	100.0		99.9		89.8		88.1	
0.5	95.4	0.999	95.9	0.955	83.8	0.995	81.8	** < 0.001**
1	94.9	0.943	95.7	0.955	83.8	0.868	81.2	** < 0.001**
2	95.2	0.890	95.7	0.687	83.7	0.868	80.3	** < 0.001**
3	94.0	0.066	93.7	0.270	83.4	0.133	79.9	** < 0.001**
4	93.1	**0.030**	93.6	**0.012**	82.9	**0.025**	79.5	** < 0.001**
5	92.8	**0.003**	93.5	**0.007**	82.6	**0.017**	78.6	** < 0.001**
7.5	93.2	0.13	93.3	** < 0.001**	81.2	** < 0.001**	77.5	** < 0.001**
15	91.6	** < 0.001**	92.6	** < 0.001**	78.0	** < 0.001**	77.0	** < 0.001**

*The FE average is either a mean FE value if FE variables at all particle sizes (0.3–5 µm) passed the normality test or a median FE value if FE variables failed the normality test.

For the KN95 masks, the FE at PSs of 2 and 5 µm was almost stable (FE98.3-100%) following GIR (Fig. 2-A, left panel). However, a gradual decrease in FE at PSs between 0.3 and 1 µm was observed to reach 81.5% following 15 Gy GIR. Statistically, the KN95 masks showed an overall significant reduction (*P* < 0.001) in FE with increased GIR doses. In particular, the FE of irradiated KN95 masks at 0.3, 0.5, 0.7 and 1 µm was significantly lower than at 5 µm (*P* ≤ 0.001). Similarly, FE of irradiated KN95 masks at 0.3 0.5, 0.7 and 1 µm was significantly lower than 2 µm (*P* ≤ 0.016). Furthermore, FE of irradiated KN95 masks at 0.3 and 0.5 µm, overall GIR doses, was significantly lower than FE at 0.7 and 1 µm (*P* ≤ 0.002).

For the N95-3M masks, the FE at PS of 5 µm was more stable (FE94.3-98.7%) than other PSs that showed a progressive decrease to reach 62.9% following 15 Gy GIR (Fig. 2-B, left panel). Statistically, the N95-3M masks showed an overall significant reduction (*P* < 0.001) in FE with increasing GIR doses. In particular, FE of irradiated N95-3M masks at each PS was significantly different from another (*P* ≤ 0.002).

#### Filtration efficiency (FE) of FFRs following low dose rate gamma irradiation

The highest decreases in FE of KN95 and N95-3M masks were observed following 15 kGy at small PSs of ≤ 0.7, with percentage decreases of 11.6% and 22.8%, respectively (Supplementary Table S1). Statistically, the average FE values of the KN95 masks were significantly lower than control (0 kGy) at GIR doses of 4, 5, 7.5 and 15 kGy (One Way Repeated Measure Analysis of Variance, *P* ≤ 0.012), whereas the average FE values of N95-3M masks were significantly lower than control at all GIR doses (*P* < 0.001; Table 1).

For the KN95 masks, the FE at PSs of 2 and 5 µm was almost stable (FE = 98.6–99.9%) following GIR (Fig. 2-A, right panel). However, a decrease in FE (88.0–99.9%) at PSs between 0.3 and 1 µm was observed following GIR. Statistically, the KN95 masks exhibited an overall significant reduction (*P* < 0.001) in FE with increased GIR doses. In particular, the FE of irradiated KN95 masks at PSs of 0.3, 0.5, and 0.7 µm was significantly lower than FE at 5 µm (*P* ≤ 0.002). Similarly, FE of irradiated KN95 masks at 0.3, 0.5, and 0.7 µm, over GIR, was significantly lower than FE at 2 µm (*P*-values were 0.014, 0.020 and 0.020, respectively).

For N95-3M masks, the FE at a PS of 5 µm was more stable (FE = 94.7–98.6%) than at other PSs (FE = 60.8–95.5%) following GIR (Fig. 2-B, right panel). Statistically, the N95-3M masks showed an overall significant reduction (*P* < 0.001) in FE with increased GIR doses. In particular, FE of irradiated N95-3M masks at 0.3, 0.5, 0.7, 1 and 2 µm was significantly lower than FE at 5 µm (*P* ≤ 0.001). Similarly, FE of irradiated N95-3M masks at 0.3, 0.5, 0.7 and 1 µm was significantly lower than FE at 2 µm (*P* ≤ 0.001). Furthermore, FE of irradiated N95-3M masks at 0.3 and 0.5 µm, over all GIR doses, was significantly lower than FE at 0.7 and 1 µm (*P* ≤ 0.001).

Taking both dose rates together, the KN95 masks following GIR showed higher FE (81.5–100%) than 3M-N95 masks (FE = 60.8–98.6%) (Fig. 2). The FE of both masks at large PSs (2 and 5 µm) was somewhat stable compared to FE at small PSs (0.3–1 µm). In general, the FE values were decreased with the increased GIR doses. The highest decreases in FE of KN95 and N95-3M masks were observed following 15 kGy, with percentage decreases ranging between 0.60 and 22.8% (Supplementary Table S1).

For both dose rates, the average FE values of KN95 masks irradiated with 4, 5, 7.5 and 15 kGy were significantly (*P* ≤ 0.03) lower than control (0 kGy), whereas the average FE of N95-3M masks following all GIR doses (0.5–15 kGy) were significantly lower than control (*P* ≤ 0.025). Interestingly, no significant differences (*P* > 0.05) were observed between typical and low dose rates GIR regarding their effects on FE of both masks.

**Figure 3:**
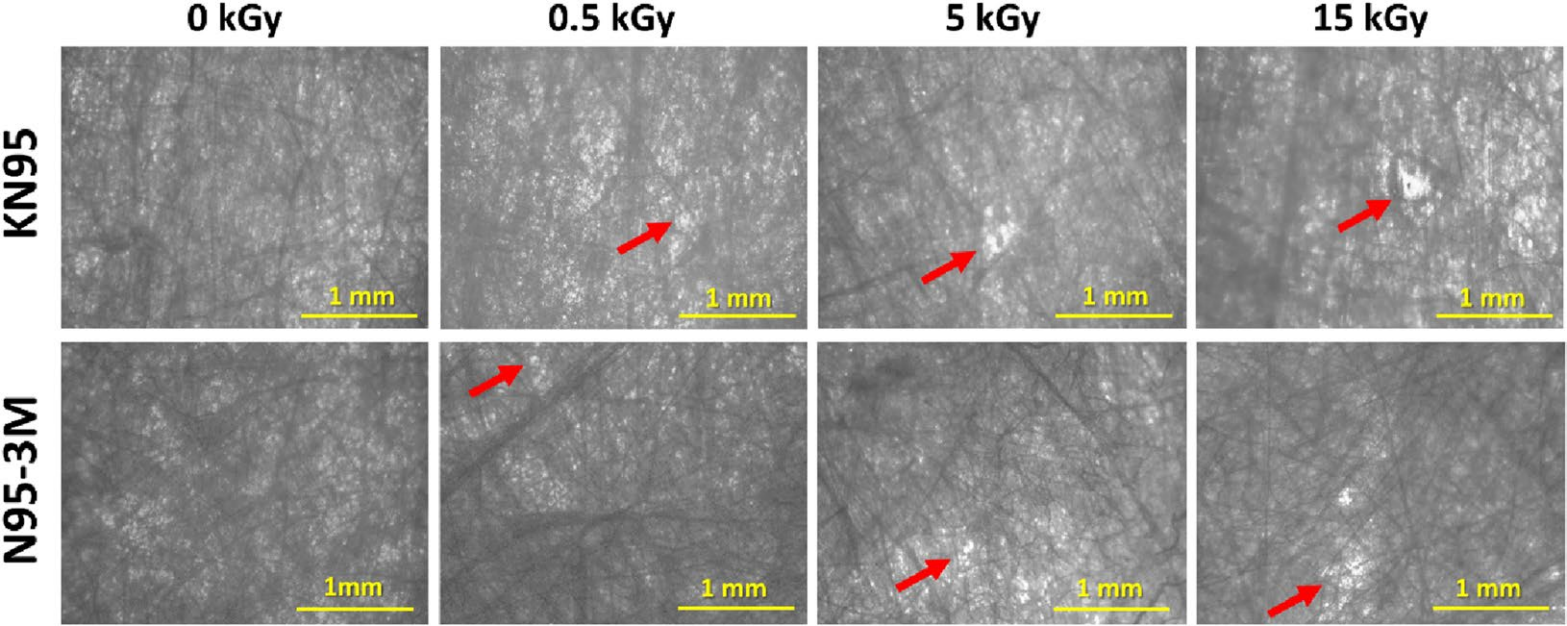
Representative microscopic images of KN95 and N95-3 M masks showing fabrics after 0, 0.5, 5 and 15 kGy low dose rate gamma irradiation (0.5088 kGy/hour). Arrows indicate physical changes (e.g. holes) within masks’ fabrics resulting from alteration in polymer homogeneity following irradiation. Photomicrographs are at 5 × magnification where scale bars show 1 mm.

In terms of evaluating the effect of low dose rate GIR on the physical structure of FFRs, no signs of visible changes or damages were observed. However, microscopic images (using ZEISS Axio Vert.A1 microscope, ZEISS, Germany) of the fiber layers of both masks were snapped following 0, 0.5, 5 and 15 k Gy. The images showed some unusual micro holes in the irradiated fabrics compared to the control (Fig. 3). These holes may have resulted from alterations in polymer homogeneity caused by GIR, such as polymer clustering, cracking, and degradation, which may explain the instability of both masks at small PSs ≤ 1 µm following GIR.

### SARS-CoV-2 RNA detection using RT-qPCR assay

#### *SARS-CoV-2 RNA stability following incubation at room temperature (RT) versus − *80* °C*

**Figure 4:**
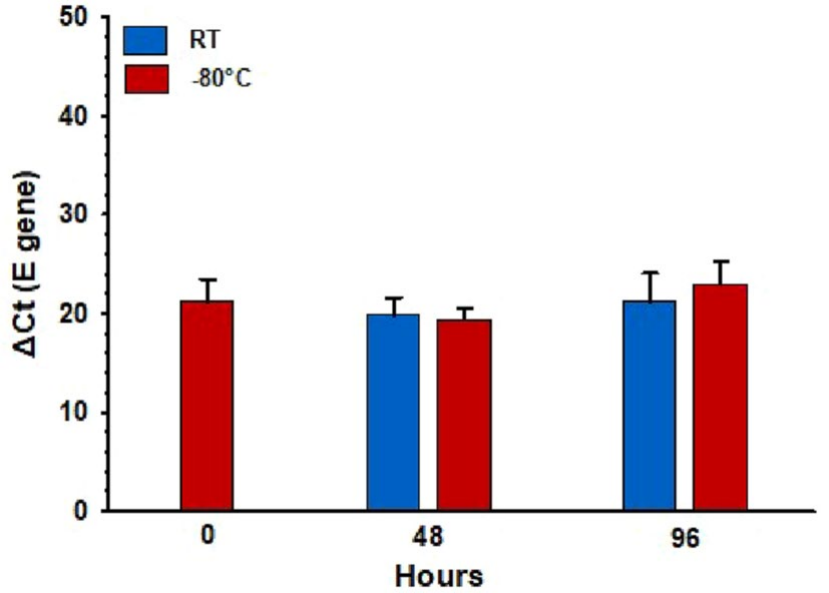
Effect of incubation time at room temperature (RT) and − 80 °C on the detection of SARS-CoV-2 RNA E gene. No significant difference was observed between incubation at RT versus − 80 or between different incubation times (*P* ≥ 0.293). ΔCt: delta cycle threshold in RT-qPCR test. Bars represent the mean and error bars indicate the standard deviation.

Results showed that the viral E Gene RNA was detected in all samples at mean cycle threshold (ΔCt) values between 19.3 and 22.9 (Fig. 4). Interestingly, incubation of SARS-CoV-2 RNA samples at RT, as compared to − 80 °C, for 48 and 96 h had no significant effect on RNA detectability (T-test, two-tailed *P*-values = 0.713 and 0.467, respectively). In addition, there were no significant differences in Ct values between 0, 48 and 96 h (RT along with − 80 °C) of incubation time (*P* = 0.293, parametric one-way repeated measures analysis of variance “RM-ANOVA”).

#### SARS-CoV-2 RNA stability following gamma irradiation

**Figure 5:**
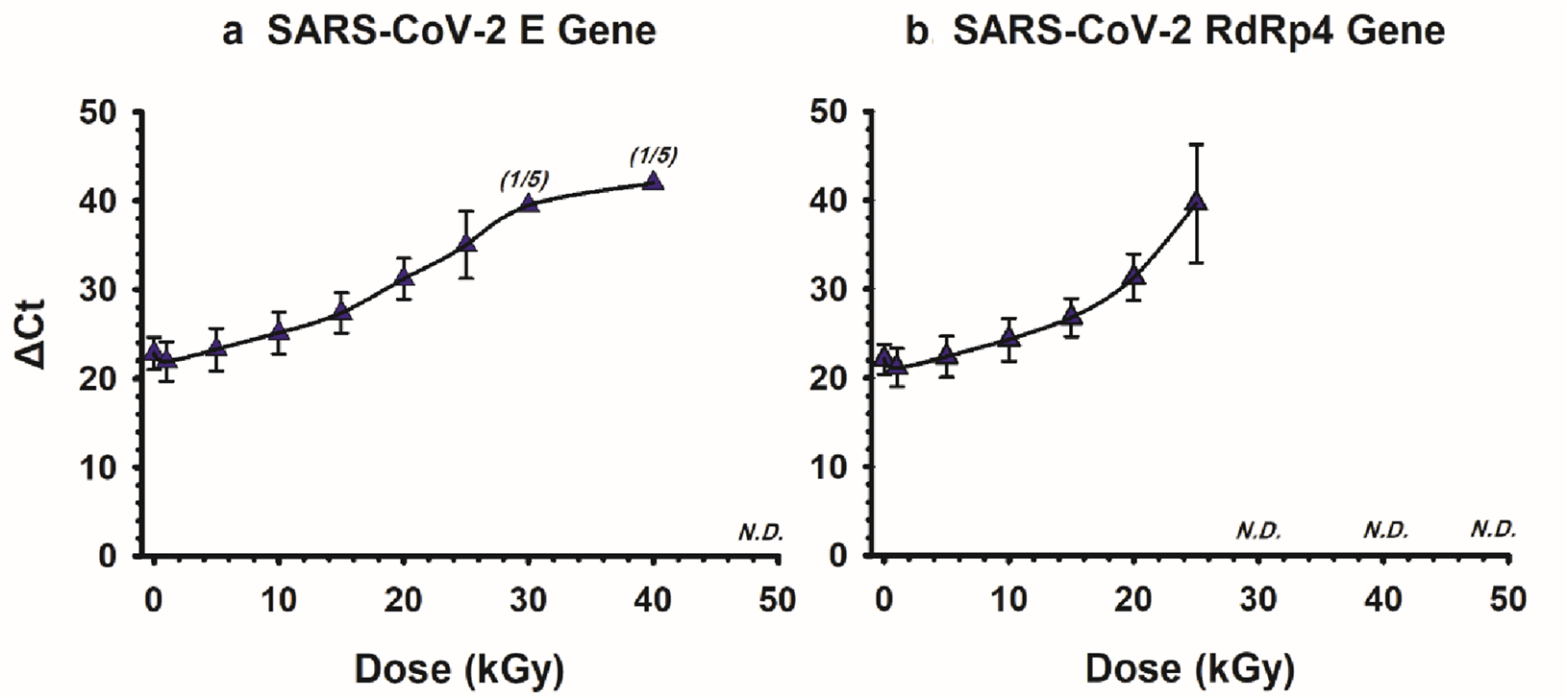
Delta Ct values of SARS-CoV-2 RNA samples irradiated with gamma irradiation. Five samples were treated with 1, 5, 10, 15, 20, 25, 30, 40 and 50 kGy. Detection of SARS-CoV-2 RNA was performed using RT-qPCR based on both E (**a**) and RdRp4 genes (**b**). Statistically, both genes showed an overall significant (*P* < 0.001) increase in ΔCt values with increased doses of gamma radiation. Pairwise comparison for E gene (0 kGy vs. 15, 20, 25, 30 and 40 kGy) were significant (*P* < 0.001). Pairwise comparison for RdRp4 gene (0 kGy vs. 20 and 25 kGy) were significant (*P* < 0.001). 1/5: one sample out of 5 was detected. N.D.: not detected. Symbols represent the mean and error bars indicate the standard deviation.

**Figure 6:**
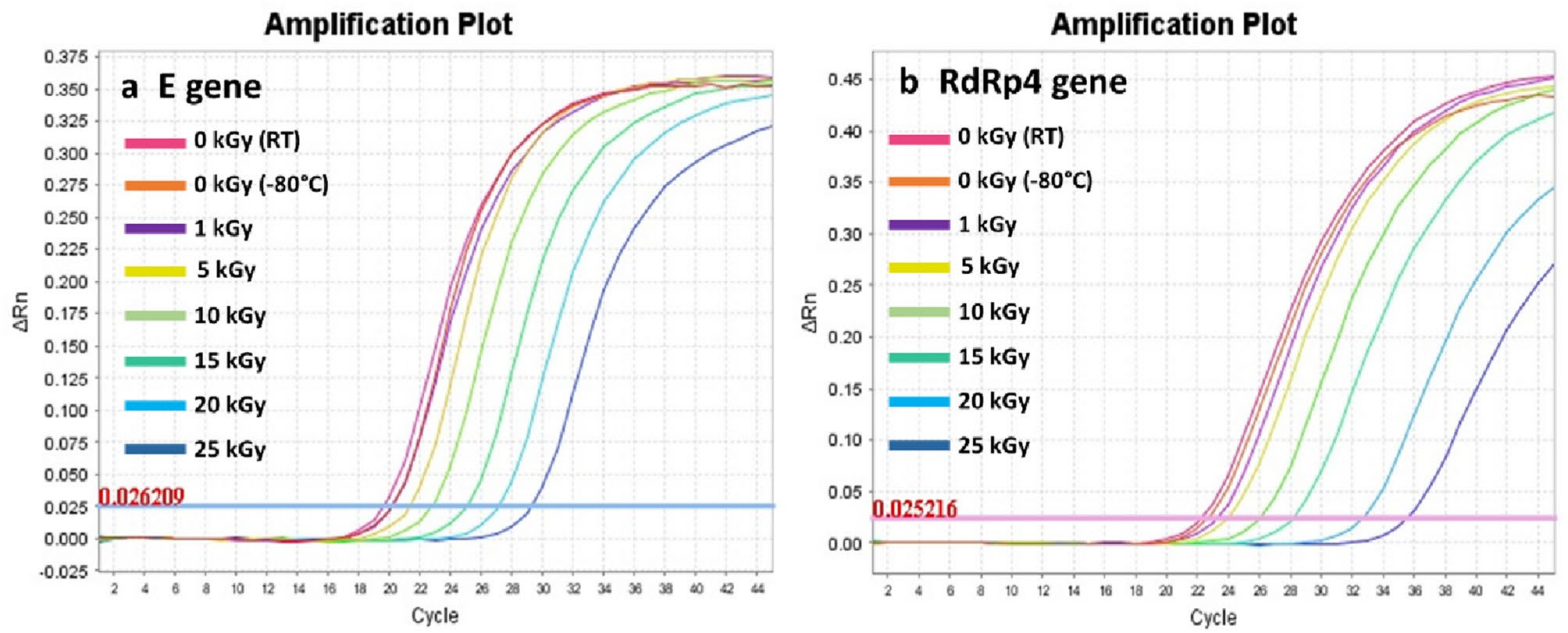
Representative images of RT-qPCR results of SARS-CoV-2 RNA E (**a**) and RdRp4 (**b**) genes detection following different gamma irradiation doses ranging from 1 to 25 Gy.

The effect of the GIR (0.5088 kGy/h) doses (0 to 50 kGy) on the detectability of SARS-CoV-2 RNA was assessed in 2 viral genes, envelope (E) and RNA dependent RNA polymerase 4 (RdRp4). Results of the respective ΔCt values are presented in Fig. 5. Representative images of RT-qPCR results for both genes are presented in Fig. 6. Obviously, the ΔCt detection threshold increased with increasing irradiation doses, indicating progressive radiation-induced degradation of viral RNA. For both genes, the RM-ANOVA indicated an overall statistically significant difference (*P* < 0.001) in the mean ΔCt values between the different radiation doses.

Based on the E gene detection, results of the pairwise multiple comparison (Bonferroni t-test) showed that GIR at doses of 15 (ΔCt = 27.4), 20 (ΔCt = 31.2), 25 (ΔCt = 35.0), 30 (ΔCt = 39.5) and 40 (ΔCt = 42.0) kGy exhibited a significant (*P* ≤ 0.001) increase in the mean ΔCt values compared to control (ΔCt = 22.8). Meanwhile, no significant difference (*P* > 0.22) was observed between 1 (ΔCt = 21.9), 5 (ΔCt = 23.3) and 10 (ΔCt = 25.1) as compared to the control 0 Gy. Note that the SARS-CoV-2 RNA E gene was only detected in 1 out of 5 samples (i.e. 20%) following 30 (ΔCt value of 39.5) and 40 (ΔCt value of 42.0) kGy, with no detection was observed at 50 kGy (Fig. 5-A).

Likewise, the results of the pairwise multiple comparison (Bonferroni t-test) of RdRp4 gene detection showed that GIR at doses of 20 (ΔCt = 31.3) and 25 (ΔCt = 39.6) kGy caused a statistically significant (*P* < 0.001) increase in the mean ΔCt values compared to 0 Gy control (ΔCt = 22.1). Meanwhile, no significant difference (*P *> 0.137) was observed between 1 (ΔCt = 21.2), 5 (ΔCt = 22.4), 10 (ΔCt = 24.3) and 15 (ΔCt = 26.8) as compared to the control 0 Gy. Furthermore, the RdRp4 gene was not detected in all 5 samples following 30, 40 and 50 kGy (Fig. 5-B).

## Discussion

We have evaluated the FE of N95-3M and KN95 following GIR as a potential decontamination method for subsequent reuse of FFRs as a crisis strategy in case of N95 masks shortage^3^. The results showed that the KN95 mask retained an initial FE of ≥ 99.7% at a PS of 0.3 µm or larger, whereas the N95-3M mask showed a range of FE between 77.1% at 0.3 µm and 98.7% at 5 µm (Fig. 1). The overall means FE of KN95 masks were significantly higher (*P* = 0.022) than N95-3M masks. This agrees with a recent study showing an average KN95 FE of 96.7% at PS between 0.03 and 0.5 µm^33^. In reasonable agreement with our results, Cramer et al. found that control N95-3M masks displayed an initial FE of 86.6%, 88.4% and 87.7% at PSs of 0.3, 0.5 and 1 µm, which were 77.1%, 79.7% and 90.9%, respectively in our results^6^. It is worth pointing out that the latter study used the same FE evaluation parameters assessment used in our study in terms of using the PM air as a source of the particles, using the same AeroTrak particle counter (TSI, Model 9306) as a particle counter and using the same air filter face velocity (0.4 m/s).

The current study hypothesized that the adverse effect of GIR on FFRs could be minimized by using low dose rate GIR. This was suggested from the literature where a potential association between increasing GIR dose rate and decreasing FE^6,16–19^. Furthermore, a recent consultancy meeting on “radiation effects on polymer materials” organized by The International Atomic Energy Agency (IAEA) recommended expanding the research on the GIR dose rate and its potential impact on minimizing polymer alterations, stating that “it was determined that published studies on dose rate effects on polymers are largely lacking”^34^. Our results showed that the FE of KN95 masks was almost stable at PSs of 2 and 5 µm following GIR of either typical or low (FE = 98.3–100.0%) dose rates (Fig. 2-A). However, a decrease in FE of KN95 masks at PSs between 0.3 and 1 µm was observed following typical or low dose rates, reaching 88.0% and 81.5%, respectively. For N95-3M masks, our results showed that the FE value at PSs of 5 µm (94.3–98.7%) was more stable than other PSs following typical or low dose rates GIR (Fig. 2-B). Overall, no significant differences (*P* > 0.05) were observed between typical and low dose rates GIR in terms of their effects on FE of each mask.

This is the first study to investigate the effect of GIR on KN95 masks as well as the first to compare the effect of different dose rates of GIR on FE of FFRs. Only five studies, with two of them are preprinted manuscripts, investigated the effect of GIR on FFRs using different evaluation parameters including GIR dose rate, particle size, air filter face velocity, flow rate, particle counter and source of particles (Table 2)^6,16–19^. In disagreement with our findings, Cramer and colleagues observed a 67.0%, 54.5% and 22.1% decrease in FE of N95-3M masks, at PSs of 0.3, 0.5 and 1 µm following 1 kGy (2.2 kGy/h)^6^. In comparison, there were only 9.9%, 10.1% and 6.9% for the typical dose rate and 7.7%, 11.0%, and 8.6% for the low dose rate, respectively (Supplementary Table S2) in our study. It is worthwhile noting that the GIR dose rate used was higher (2.2 kGy/h) than those used in our study. Another study also showed a ~ 50% decrease in FE at PS of 0.075 µm of two other FFRS types following a single dose of 25 kGy (1.8 kGy/h), a dose that was not examined in our study^19^. Furthermore, the study showed that GIR exhibited significant changes in electrostatic charges of the filtration layer within the FFRs.

**Table 2:** Comparison between the parameters used in five studies investigating the filtration efficiency of FFRs following gamma irradiation.

Study	Radiation Dose (kGy)	Dose rate (kGy/h)	particle size (µm)	filter face velocity (m/s)	Flow rate (L/min)	Ambiance (aerosol vs PM air)	Particle counter	Reference
1	1, 10 and 50	2.2	0.1, 0.5 and 1	0.4	2.8	APM	Aerotrak 9306 (TSI)	^6^
2**	15 and 25	1.94	0.3, 0.5 and 1	0.5 and 2.6	20 and 90	Aerosol	OPC*	^16^
3**	15 and 25	1.94	0.3,0.5, 1, 3, 5 and 10	0.50	20 and 90	Aerosol	OPC*	^17^
4	15 and 25	1.94	0.3, 0.5, 1 and 5	0.50	20 and 90	Aerosol	OPC*	^18^
5	25	1.8	0.075–0.3***	0.17	100	Aerosol	Aerotrak 8020 (TSI)	^19^
The current study	0.5, 1,2,3,4,5,7.5 and 15	1.65 and 0.5088	0.3, 0.5, 0.7, 1, 2 and 5	0.4	2.8	APM	Aerotrak 9306 (TSI)	*NA*

*Optical Particle Counter (Model 1.108, Grimm Aerosol Technik, Germany). **Preprinted study. ***Continuous measurement.

Furthermore, three reports showed mild adverse GIR effect (< 50% decrease in FE) on FFRs, which are comparable with our findings^16–18^. The first preprint showed that the average FE of N95 masks at ≥ 0.3 µm was decreased by 29.6% following 15 kGy, while it was 12.9% in our results (Supplementary Table S1)^16^. The second preprint showed a ~ 28% decrease in FE at 0.3 µm following 15 kGy, while it was 19.75% in our findings^17^. However, the latter preprint showed no effect of GIR on FE at 5 µm, which differs from our findings as ~ 4.5% decreases in FE was observed (Supplementary Table S1). The third preprint showed that GIR exhibited a 3.7% decrease in FE at 1 µm following 15 kGy, while it was 11% (9.6–12.4) in our results^18^. Taken all the five studies together, GIR had an adverse effect on FE of FFRs although they showed variations in FE data. These variations may be explained by the influence of various factors on the FE assessment such as FE evaluation parameters, GIR dose rate and FFR types.

To try to minimize the adverse effect of GIR on the fabrics of FFEs, two suggestions related to irradiation conditions could help preserve FFRs efficiency. The first suggestion is to irradiate the FFRs in a free-oxygen (e.g. nitrogen or vacuum) container^34,35^. The structural destruction of the PP (the main component of the FFRs filter fabrics) following GIR is particularly noticeable in the presence of air due to the oxidative damage of the PP’s structure^12^. However, applying this idea should take into consideration that the structural components of viruses, such as nucleic acids, protein and lipid, could be more resistant to radiation in the anoxic than aerobic conditions^36,37^. The second suggestion is to irradiate the FFEs at a low-temperature atmosphere to decrease PP cross-linking within FFEs during the irradiation process^38^. Although cold-irradiation processing may protect against PP damage, it can also limit damage to the pathogens, the target of the irradiation process^39,40^.

Although our observation did not notice any visible changes in the FFRs structure following GIR, microscopic changes were observed within masks’ materials, which may explain the FE instability following GIR (Fig. 3). These changes were expected as several studies reported that GIR exhibited significant physical changes in the PP, the main component in the filtration layers of FFRs^12–15^. Therefore, further monitoring of the potential changes in physical structure and electrostatic charge status of decontaminated FFRs should be considered. In terms of evaluating the fit factor of the irradiated FFRs, previous studies showed that GIR has no effect on the fit factor of FFRs although it degrades FFRs’ FE^6,16,17^. In addition, a recent IAEA report agreed with the latter studies and showed that 24 kGy has no effect on the fit factor on irradiated FFRs^41^.

Interestingly, results showed that the viral E Gene RNA was detected at RT up to 96 h, with no significant difference compared to − 80 °C incubation (Fig. 4). Our data also showed that E and RdRp4 SARS-CoV-2 genes (Fig. 5) could be constantly detected up to 25 kGy, and randomly at 30–40 kGy, but not at 50 kGy. Two reports so far evaluated the ability of GIR to inactivate SARS-CoV-2 using the median tissue culture infectious dose (TCID_50_) assay^30,31^. Both studies found that the cultured active SARS-CoV-2 was completely inactivated by GIR at the dose of 10 kGy. However, Leung et al. found that the E and the nucleocapsid (NP) SARS-CoV-2 genes can still be detected following 50 kGy using RT-qPCR assay^30^. The large difference between the GIR dose needed to observe complete viral inactivation (10 kGy) and to achieve complete viral RNA degradation (50 kGy) may emanate from the inherent characteristics of each assay. Although SARS-CoV-2 genes were still detectable at 40 kGy by RT-qPCR, it does not necessarily mean that RNA was intact, and the viral particle of SARS-CoV-2 was still infectious. The influence of GIR on other biological factors rather than RNA, such as protein and lipids, seems limited as a study showed that the integrity of viral morphology and protein structures of coronaviruses were preserved following 10 kGy^42^.

In conclusion, the KN95 mask showed a higher initial FE than the 3M-N95 masks. Irradiated KN95 and N95-3M masks showed microstructural changes within masks’ fabrics associated with dose-dependent substantial reductions (≤ 18.5%) in FE at small particle sizes (0.3–2 µm) and moderate reductions (≤ 4.5%) at large (5 µm) particle size, the size of the most suspected droplets implicated in COVID-19 transmission. GIR dose rate does not seem to be an influencing factor on the FE of irradiated FFRs. Incubation for 4-days at room temperature has no effect on SARS-CoV-2 detectability using RT-qPCR assay. The mean cycle threshold (ΔCt) of viral RNA detection increased with increasing GIR doses with an absence of detection at very high doses.

## Materials and methods

Authors confirm that all methods were carried out in accordance with relevant guidelines and regulations.

### Filtering facepiece respirators (FFRs)

This study used two FFRs types: KN95 and 3M-N95 masks. The KN95 masks (molded shape) were manufactured by ZhongShan XiaoLan YiShuai Garment Factory, Zhongshan, China, with a lot number of A12199, and have met the Chinese standards for FFRs^43^. The 3M-N95 NIOSH-approved masks were manufactured by 3M North American Company with a model number of 8210 and lot number of A12199^2^. Although NIOSH has not approved KN95 masks to be used as FFRs, it recommended the KN95 to be used in the medical settings when NIOSH-approved masks are unavailable^44^.

### Gamma irradiation sources

This study used two GIR sources representing two different dose rates: typical and low. The first GIR source is a typical dose rate source located at the Nuclear Science Research Institute, King Abdulaziz City for Science and Technology (KACST), Riyadh, Saudi Arabia. The institute uses a Gamma Cell 220 cobalt-60 source (MDS Nordion, Ottawa, Canada) with a dose rate of 1.65 kGy/h. The second GIR source is an industrial low dose rate source located at the Gamma Irradiation Facility (GIF), Biomedical Physics Department, King Faisal Specialist Hospital and Research Centre, Riyadh, Saudi Arabia. The GIF uses a cobalt-60 gamma source (Puridec Irradiation Technology, CHESHAM, Buckinghamshire, England) with a current dose rate of 0.5088 kGy/h. The typical GIR dose rate used in FFRs decontamination ranges between 1.8 and 2.2 kGy/h^6,16–19^. The first (typical dose rate) GIR source was used to irradiate FFRs (0.5–15 kGy) only, while the second (low dose rate) GIR source was used to irradiate FFRs (0.5–15 kGy) and SARS-CoV-2 RNA samples (1–50 kGy). The GIR doses ranged from 0 to 50 kGy and the required time needed to deliver the doses extended up to 98.16 h for the highest dose.

### Filtration efficiency (FE) assessment of filtering facepiece respirators (FFRs)

#### Filtration efficiency (FE) measurement

The FE measurement used in this study is an in-house method that was previously described^45^. Briefly, a custom-designed air duct was manufactured to measure the FE of different FFRs types using the PM air as a source of the measured particles. The overall dimensions of the air duct are 19-cm-long, 14-cm-wide and 12-cm-high. The air duct consists of two parts, head and tail, that can be tightly joined together by three mold bolts to squeeze a filter in a sandwich manner, with no air leak present between both parts. The head of the air duct is connected to an AeroTrak particle counter (TSI, Model 9306) that counts particles with sizes of 0.3, 0.5, 0.7, 1, 2 and 5 µm at a flow rate of 2.8 liters/minute (L/min). The tail of the air duct is connected to an electrical fan that flows the PM air through the air duct tail to give a face velocity of 0.4 m/s; measured by Velocicalc Air Velocity Meter 9545 (TSI, product ID# 9545-A).

#### Initial filtration efficiency (FE) of FFRs

A total of 10 FFRs of each type (N95-3M and KN95) were subjected to FE evaluation. The 10 FFRs will be used later as controls (0 kGy) for FFRs irradiated with either low dose GIR (5 FFRs) or typical dose rate GIR (5 FFRs).

The particle number concentration of the PM air was assessed at least 5 times before FFRs assessment. For each PS, FE was calculated using the following formula:

$$ FE \left( \% \right)\, = \,100 - \left( {\frac{number\,of\,penetrated\,particles }{{average\,number\,of\,particles\,in\,air}}\, \times \,100} \right) $$

#### Filtration efficiency (FE) of FFRs following gamma irradiation

For each source of GIR (typical and low dose rates), 5 FFRs of each type (KN95 and N95-3M) were exposed to 8 different radiation doses (0.5, 1, 2, 3, 4, 5, 7.5 and 15 kGy) with 0 kGy as a control. In total, 45 masks of each type of FFRs were used in the study.

### Detection of SARS-CoV-2 RNA following gamma irradiation

The stability of 5 SARS-CoV-2 RNA samples against GIR doses (0–50 kGy) was evaluated using the RT-qPCR assay targeting the E and RdRp4 SARS-CoV-2 genes.

#### SARS-CoV-2 samples collection and ethical considerations

Nasopharyngeal swabs were obtained from COVID-19 patients between May and July 2020. The samples were collected for diagnosis and archived for research purposes. The Institutional Review Board (IRB) at King Faisal Specialist Hospital and Research Centre (KFSH&RC), Riyadh, Saudi Arabia (RAC Approval# 2200031) has approved the study. Five SARS-CoV-2 positive samples were randomly retrieved, anonymized, coded and used in this study. The IRB granted a waiver for obtaining informed consent owing to the use of anonymized archived samples.

#### Samples processing and RNA extraction

The five nasopharyngeal swabs were submerged in viral transport medium for diagnostic analysis in the microbiology laboratory at the Department of Pathology, KFSH&RC, Riyadh, Saudi Arabia. Then, aliquots of leftover samples were stored at – 80 °C until viral RNA extraction in a biosafety level-3 research laboratory. Viral RNA extraction was performed using an in-house automated RNA extraction protocol^46^.

#### *SARS-CoV-2 RNA stability following incubation at room temperature (RT) versus − *80 °C

As both GIR facilities used do not have cooling systems, pre-irradiation optimization experiments were performed to evaluate the effect of RT incubation (0, 48 and 96 h) on the stability of SARS-CoV-2 RNA using RT-qPCR assay. Three SARS-CoV-2 RNA samples were thawed from − 80°C, then each sample was aliquoted into 3 test tubes, and incubated for 0, 48 or 96 h. RT-qPCR assay (E gene) was performed directly after each time point for SARS-CoV-2 RNA detection.

#### Effect of gamma irradiation on SARS-CoV-2 RNA detection

Each of the 5 RNA samples of COVID-19 patients were individually aliquoted into a total of 11 tubes; 2 tubes were allocated for controls without irradiation (0 kGy) kept either at RT or at – 80 °C for the duration of the experiment, while 9 tubes were exposed to 9 different low dose rate GIR doses (1, 5, 10, 15, 20, 25, 30, 40 and 50 kGy). This experiment was initially performed with irradiation doses of 1, 5, 10, 15, 20 and 25 kGy. As SARS-CoV-2 E and RdRp4 genes were still detectable at 25 kGy dose, we have added 30, 40 and 50 kGy along with two samples (RT and – 80 °C) were added as controls.

#### Reverse transcription quantitative real-time polymerase chain reaction (RT-qPCR)

The TAQPATH COVID-19 CE-IVD RT-PCR reverse transcription quantitative real-time polymerase chain reaction kit (Thermo Fisher Scientific: A48102) was used as described previously^47^. The primers of E and RdRp4 SARS-CoV-2 genes were selected to examine the presence of SARS-CoV-2 RNA based on WHO and CDC recommendations for patients’ testing and diagnosis (Supplementary Table S3), which were adapted from Charité Institute of Virology, Pasture Institute, Paris**,** France^48^. The RT-qPCR cycling conditions, the final concentrations of the reaction components in the master mix used in the study are summarized in Supplementary Tables S4 and S5, respectively.

### Statistical analysis

The paired t-test was used to examine the overall statistical differences in the initial FE between KN95 and N95-3M masks. The t-test was applied to test for significant differences in the initial FE between various PSs within each mask as well as to examine the statistical differences in the mean ΔCt values between RNA samples following RT and – 80 °C incubations. The parametric one-way repeated measures analysis of variance (RM-ANOVA) was used to test significant differences in the mean FE between irradiated and non-irradiated masks, as well as in the mean ΔCt values between irradiated and non-irradiated CoV-SARS-2 RNA samples. The Bonferroni t-test was used to correct for the pairwise multiple comparisons in the RM-ANOVA when appropriate. All the statistical Analyses were performed using SigmaPlot version 14.5 for Windows (SPSS, Chicago, USA). A *P*-value < 0.05 is considered statistically significant.

## Supplementary Information

Supplementary Information.
