# Supplementary material for: Effect of gamma irradiation on filtering facepiece respirators and SARS-CoV-2 detection
Source: Sci Rep. 2021 Oct 6;11:19888. doi: 10.1038/s41598-021-99414-6 (PMC8494839; doi:10.1038/s41598-021-99414-6)
Supplement: Supplementary file 1 — Supplementary Information. [file 41598_2021_99414_MOESM1_ESM.docx]

**Supplementary Materials:**

**Supplementary Table S1.** Filtration efficiency of KN95 and N95-3M masks before and after gamma radiation dose of 15 kGy. Abbreviation: StEr = standard error. FE% reduction = Initial (0 kGy) FE% - FE% after GIR.

|  |  | **KN95** | | | |  | **N95-3M** | | | |
| --- | --- | --- | --- | --- | --- | --- | --- | --- | --- | --- |
|  |  | **Typical dose rate** | | **Low dose rate** | |  | **Typical dose rate** | | **Low dose rate** | |
| **Particle size** |  | **0 kGy** | **15 kGy** | **0 kGy** | **15 kGy** |  | **0 kGy** | **15 kGy** | **0 kGy** | **15 kGy** |
| **0.3 µm** | FE% (StEr) | 99.9 (0.00) | 86.6 (2.09) | 99.0 (0.03) | 89.5 (0.51) |  | 76.9 (0.30) | 62.9 (0.20) | 77.3 (0.28) | 60.9 (1.00) |
|  | FE% reduction |  | 13.3 |  | 10.4 |  |  | 18.2 |  | 21.3 |
| **0.5 µm** | FE% (StEr) | 100 (0.00) | 81.5 (3.67) | 99.9 (0.02) | 88.5 (0.92) |  | 80.5 (0.24) | 66.2 (0.21) | 78.7 (0.87) | 60.8 (0.59) |
|  | FE% reduction |  | 18.5 |  | 11.4 |  |  | 17.8 |  | 22.8 |
| **0.7 µm** | FE% (StEr) | 100 (0.00) | 89.2 (2.39) | 99.6 (0.13) | 88.0 (1.47) |  | 87.9 (1.16) | 75.7 (1.47) | 88.3 (0.93) | 78.5 (0.45) |
|  | FE% reduction |  | 10.8 |  | 11.6 |  |  | 13.8 |  | 11.1 |
| **1.0 µm** | FE% (StEr) | 100 (0.00) | 94.0 (1.59) | 99.9 (0.05) | 95.8 (0.33) |  | 91.7 (0.35) | 80.4 (0.73) | 90.5 (0.25) | 81.8 (1.14) |
|  | FE% reduction |  | 6.0 |  | 4.1 |  |  | 12.4 |  | 9.6 |
| **2.0 µm** | FE% (StEr) | 100 (0.00) | 98.6 (0.34) | 99.9 (0.03) | 98.7 (0.29) |  | 96.4 (0.43) | 82.0 (0.76) | 95.5 (0.41) | 85.5 (0.48) |
|  | FE% reduction |  | 1.4 |  | 1.2 |  |  | 14.9 |  | 10.4 |
| **5.0 µm** | FE% (StEr) | 100 (0.00) | 99.4 (0.29) | 99.9 (0.14) | 98.9 (0.40) |  | 98.7 (0.44) | 94.3 (0.50) | 98.6 (0.43) | 94.7 (1.17) |
|  | FE% reduction |  | 0.6 |  | 1.0 |  |  | 4.5 |  | 4.0 |

**Supplementary Table S2.** Filtration efficiency of KN95 and N95-3M masks before and after gamma radiation dose of 1 kGy. Abbreviation: StEr = standard error. FE% reduction = Initial (0 kGy) FE% - FE% after GIR.

|  |  | **KN95** | | | |  | **N95-3M** | | | |
| --- | --- | --- | --- | --- | --- | --- | --- | --- | --- | --- |
|  |  | **Typical dose rate** | | **Low dose rate** | |  | **Typical dose rate** | | **Low dose rate** | |
| **Size** |  | **0 kGy** | **1 kGy** | **0 kGy** | **1 kGy** |  | **0 kGy** | **1 kGy** | **0 kGy** | **1 kGy** |
| **0.3 µm** | FE% (StEr) | 99.9 (0.00) | 88.4 (1.05) | 99.0 (0.03) | 91.5 (0.48) |  | 76.9 (0.30) | 69.2 (0.88) | 77.3 (0.28) | 71.4 (0.68) |
|  | FE% reduction |  | 11.6 |  | 8.4 |  |  | 9.9 |  | 7.7 |
| **0.5 µm** | FE% (StEr) | 100 (0.00) | 8.6 (2.37) | 99.9 (0.02) | 92.0 (0.75) |  | 80.5 (0.24) | 72.3 (1.31) | 78.7 (0.87) | 70.0 (1.10) |
|  | FE% reduction |  | 13.5 |  | 11.4 |  |  | 10.1 |  | 11.0 |
| **0.7 µm** | FE% (StEr) | 100 (0.00) | 93.9 (1.59) | 99.6 (0.13) | 94.7 (1.08) |  | 87.9 (1.16) | 82.2 (0.67) | 88.3 (0.93) | 80.6 (2.56) |
|  | FE% reduction |  | 6.1 |  | 4.9 |  |  | 6.5 |  | 8.8 |
| **1.0 µm** | FE% (StEr) | 100 (0.00) | 95.9 (1.09) | 99.9 (0.05) | 96.8 (0.12) |  | 91.7 (0.35) | 85.4 (0.27) | 90.5 (0.25) | 82.7 (0.89) |
|  | FE% reduction |  | 4.0 |  | 3.1 |  |  | 6.9 |  | 8.6 |
| **2.0 µm** | FE% (StEr) | 100 (0.00) | 99.4 (0.21) | 99.9 (0.03) | 99.4 (0.13) |  | 96.4 (0.43) | 87.0 (0.67) | 95.5 (0.41) | 86.6 (1.33) |
|  | FE% reduction |  | 0.6 |  | 0.5 |  |  | 9.7 |  | 9.4 |
| **5.0 µm** | FE% (StEr) | 100 (0.00) | 100 (0.00) | 99.9 (0.14) | 99.4 (0.23) |  | 98.7 (0.44) | 96.1 (0.67) | 98.6 (0.43) | 95.7 (0.46) |
|  | FE% reduction |  | 0.0 |  | 0.4 |  |  | 2.6 |  | 3.0 |

**Supplementary Table S3**. Genes and their primers and probes used for targeting SARS-CoV-2. Adapted from Pasteur protocol: Real-time RT-PCR assays for the detection of SARS-CoV-2, <https://www.who.int/docs/default-source/coronaviruse/real-time-rt-pcr-assays-for-the-detection-of-sars-cov-2-institut-pasteur-paris.pdf?sfvrsn=3662fcb6_2>

| **Target** | **Primer and probes** | **Sequence** |
| --- | --- | --- |
| **E gene** | E_Sarbeco_Reverse | ACA GGT ACG TTA ATA GTT AAT AGC GT |
|  | E_Sarbeco_Forward | ATA TTG CAG CAG TAC GCA CAC A |
|  | E_Sarbeco Probe | ACA CTA GCC ATC CTT ACT GCG CTT CG [5'] TAMRA [3'] BHQ-1 |
| **RdRp 4** | RdRp/nCoV_IP4-Forward | GGT AAC TGG TAT GAT TTC G |
|  | RdRp/nCoV_IP4-Reverse | CTG GTC AAG GTT AAT ATA GG |
|  | RdRp/nCoV_IP4-Probe | TCA TAC AAA CCA CGC CAG G [5'] FAM [3'] BHQ-1 |

**Supplementary Table S4.** RT-qPCR master mix components, volumes and concentrations.

| **Singleplex master mix** | **Volume (µL)** | **Final concentration** |
| --- | --- | --- |
| RNA sample | 5.0 | Avg 17.7 ng/µl |
| H_2_O PPI | 3.7 |  |
| Reaction mix 2X | 12.5 | 0.3 mM |
| MgSO_4_ (50 mM) | 0.4 | 0.8 mM |
| Forward Primer (10 μM) | 1.0 | 0.4 μM |
| Reverse Primer (10 μM) | 1.0 | 0.4 μM |
| Probe (10 μM) | 0.4 | 0.16 μM |
| SupersriptIII RT/ Platinum Taq Mix | 1.0 |  |
| Final volume | 25.0 |  |
|  |  |  |

**Supplementary Table S5.** RT-qPCR cycling conditions.

| **Cycle** | **Temperature (°C)** | **Time (minutes)** | **Number of cycles** |
| --- | --- | --- | --- |
| Reverse transcription | 55 | 20 | X1 |
| Denaturation | 95 | 3 | X1 |
| Amplification | 58 | 0.5 | X40 acquisition |
| Cooling | 40 | 0.5 | X1 |
